# Up-Regulated Vitamin D Receptor by *Pelargonium sidoides* Extract EPs^®^ 7630 Contributes to Rhinovirus Defense in Bronchial Epithelial Cells

**DOI:** 10.3390/ph14020172

**Published:** 2021-02-22

**Authors:** Michael Roth, Qingzhu Sun, Michael Tamm

**Affiliations:** 1 Pulmonary Cell Research & Pneumology, Department Biomedicine & Internal Medicine, University Hospital Basel, Petersgraben 4, CH-4031 Basel, Switzerland; sunqingzhu@nwafu.edu.cn (Q.S.); michael.tamm@usb.ch (M.T.); 2 College of Animal Science and Technology, Northwest A&F University, Yangling 712100, China

**Keywords:** rhinovirus, EPs^®^7630, vitamin D receptor, viral replication, bronchial epithelial cell

## Abstract

EPs^®^7630, extracted from *Pelargonium sidoides*, reduces the severity of viral upper respiratory tract infections. Vitamin D also improves anti-viral host defense through similar signaling pathways. This study assessed if EPs^®^7630 modifies vitamin D receptor (VDR) expression and function by human bronchial epithelial cells. Bronchial epithelial cells were incubated with EPs^®^7630 over 48 h before calcitriol stimulation and/or infection with *Rhinovirus* (RV)-16. Protein expression was determined by Western-blotting. Intracellular signaling of mitogen activated protein kinases (MAPK) was studied by chemical inhibitors. The anti-viral effect was assessed by immunofluorescence for RV-16 protein. EPs^®^7630 upregulated VDR expression through Erk1/2 MAPK and thereby increased the cell’s sensitivity to calcitriol. Compared ton untreated cells, the shift of the VDR into the nucleus at 5.3 times lower calcitriol concentration. EPs^®^7630 increased Erk1/2 MAPK signaling, but reduced p38 phosphorylation, and had no effect on Jun N-terminal kinase (JNK). EPs^®^7630 improved the anti-viral effect of vitamin D on RV-16 infection by 2.1 folds compared to vitamin D alone or to untreated cells. Furthermore, EPs^®^7630 improved the differentiation of epithelial cells by upregulating E-cadherin expression through Erk1/2. In conclusion, EPs^®^7630 increased host defense against *Rhinovirus* infection by upregulating the VDR and the differentiation of epithelial cells.

## 1. Introduction

Viral infections are the most frequent cause of respiratory ailments and present a severe health problem to children and the elderly [1,2]. EPs^®^ 7630 is an herbal drug preparation from the roots of *Pelargonium sidoides*, which effectively reduces the severity of symptoms in patients with acute bronchitis, acute sinusitis maxillaris, tonsillopharyngitis, or common cold, combined with a good tolerability [1,2,3,4].

The mechanisms by which EPs^®^ 7630 reduces viral infection were investigated in several non-clinical trials. It has been reported that EPs^®^ 7630 significantly reduced the ability of various viruses to attach to the host cells, or to prevent virus release from infected cells. In cell culture experiments, EPs^®^ 7630 inhibited the attachment of HIV-1 to human immune cells, protecting them from viral entry [5], whereas protection of cells against influenza A was mediated via inhibition of hemagglutin and neuraminidase activity [6]. In human bronchial epithelial cells, EPs^®^ 7630 reduced *Rhinovirus-16* (RV-16) replication by downregulating the expression of inducible co-stimulator (ICOS) and its ligand (ICOSL), as well as the surface calreticulin receptor, whereas host defense supporting protein β-defensin 1 and suppressor of cytokine signaling 1 (SOCS-1) were increased [7]. However, the intracellular signaling mechanism underlying the anti-viral effects of EPs^®^ 7630 remained to be defined.

For type-A and –B *Rhinoviruses*, the intracellular adhesion molecule1 (ICAM1) is a major docking protein of host cells [8], required for viral infection of different host cells [7,9]. Vitamin D receptor (VDR) activation has been shown to reduce the adherence of RV to intracellular adhesion molecule 1 (ICAM1) [10]. In this context, low serum levels of vitamin D correlated with increased susceptibility to respiratory virus infection. In children, *rhinovirus* infection inversely correlated with vitamin D levels [11], and low serum vitamin D increased the sensitivity to RV infection [12]. Vitamin D deficiency correlated with the frequency of exacerbation caused by *rhinovirus* infection in chronic obstructive pulmonary disease (COPD) patients [13]. Furthermore, infection with either RV or *Respiratory syncytial virus* (RSV) reduced the expression of the VDR by human epithelial cells, but this was reversed by vitamin D supplementation [14].

The importance of the VDR in host defense is supported by reports that the VDR genotype affects the risk of upper respiratory tract infections. In children with asthma, a genome analysis indicated that the VDR is one of the most important factors that regulates the susceptibility to virus-induced upper respiratory tract infections [15]. In a cohort of 1462 adults from the U.K., a link between the minor VDR allele, rs4334089 SNP and the susceptibility to upper respiratory tract infections was confirmed [16]. In children with severe bronchiolitis, SNP rs2228570 VDR together with SNPs of Toll like receptor 4 and Toll like receptor 2 was associated with increased risk of death [17].

The expression of the VDR is controlled by extracellular-signal regulated kinase 1/2 (Erk1/2) mitogen activated protein kinase (MAPK) in muscle cells [18]. EPs^®^ 7630 has been shown to differentially regulate Erk1/2 MAPK in immune cells, while suppressing protein 38) (p38) MAPK [19]. Hence, it seems feasible that EPs^®^ 7630 could also affect VDR expression.

In this study, we assessed the effect of EPs^®^ 7630 on the expression and activation of the VDR in bronchial epithelial cells and determined its functional relevance for the anti-viral activity.

## 2. Results

### 2.1. Analysis of EPs^®^ 7630 Components by High Pressure Liquid Chromatography (HPLC)

**Figure 1:**
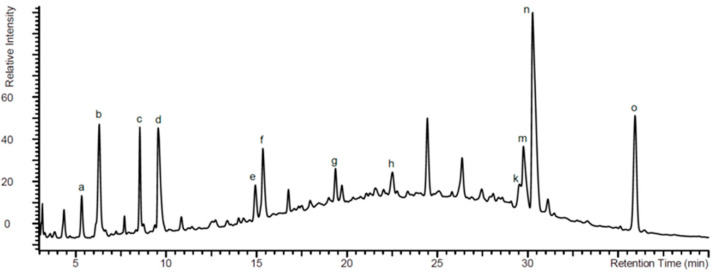
High pressure liquid chromatography (HPLC) fingerprint detected by UV at 280 nm. The major peaks were assigned by analysis of high resolution mass spectrometry (HRMS) data and are given as follows: (**a**) adenosine 3′,5′-cyclic monophosphate, (**b**) guanosine 3′,5′-cyclic monophosphate, (**c**) 1-methylguanosine 3′,5′-cyclic monophosphate, and (**d**) benzopyranones. The assigned structures are given in Table 1.

Figure 1 shows a typical HPLC-UV-HRMS profile of EPs^®^ 7630. The chemical structure of the different benzopyranones contained in EPs^®^ 7630 are assigned by small letters (d–o) and the chemical structure analysis is provided in Table 1. More details of the EPs^®^ 7630 constituents were published by others [20,21].

### 2.2. VDR Regulation by EPs^®^ 7630 and the Underlying Signaling Pathway

**Figure 2:**
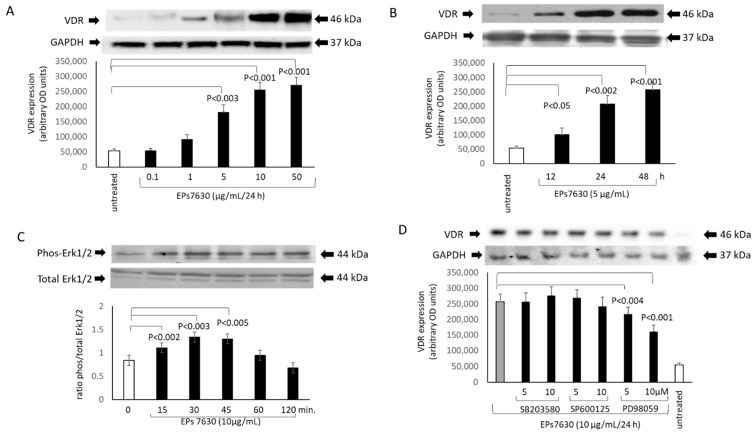
EPs^®^ 7630 modifies vitamin D receptor (VDR) expression. (**A**) Representative Western-blots and image analyses of VDR expression in primary bronchial epithelial cells (*n* = 4) stimulated with increasing concentrations of EPs^®^ 7630 over 24 h. Bars represent mean ± SEM. (**B**) Kinetic of EPs^®^ 7630 (10 μg/mL) induced VDR expression over 48 h (*n* = 4). (**C**) The effect of EPs^®^ 7630 on the phosphorylation of Erk1/2 mitogen activated protein kinases (MAPK) over 120 min. (**D**) The effect of inhibitors for Jun N-terminal kinase (JNK), p38, and Erk1/2 on EPs^®^ 7630 induced VDR expression (*n* = 4). Representative Western-blots are depicted above the corresponding bars. Similar results were obtained in BEAS-2B cells.

Epithelial cells treated with EPs^®^ 7630 increased the expression of the VDR in a concentration-dependent manner over 24 h (Figure 2A). There was no difference between primary epithelial cells or BEAS-2B cells. This effect became significant at concentration > 5 µg/mL EPs^®^ 7630 after 24 h (Figure 2A). Kinetics of the EPs^®^ 7630-induced VDR expression were studied with a fixed concentration of EPs^®^ 7630 (10 µg/mL) over 48 h which showed a continuous increase (Figure 2B).

Based on earlier reports, the involvement of MAPKs in the EPs^®^ 7630-mediated activation of the VDR expression was studied. In Figure 2C the kinetic of the stimulating effect of EPs^®^ 7630 on phosphorylation of Erk1/2 is shown. None of the other MAPKs was significantly modified by EPs^®^ 7630 (data not shown). Blocking MAPK signaling pathways by specific chemical inhibitors, the stimulating effect of EPs^®^ 7630 (10 µg/mL) on VDR expression was significantly reduced by PD98059 in a concentration dependent manner (Figure 2D). In contrast, neither the inhibition of p38 MAPK by SB203580, nor that of Jun N-terminal kinase (JNK) by SP600125 had any significant effect on EPs^®^ 7630 induced VDR expression (Figure 2D).

**Figure 3:**
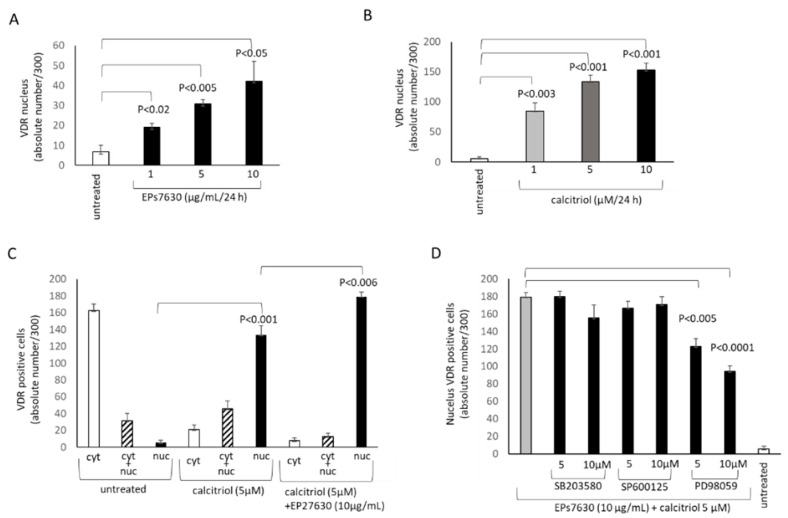
EPs^®^ 7630 and calcitriol activate the VDR. (**A**) Concentration dependent increase of nuclear VDR by EPs^®^ 7630 over 24 h in primary human bronchial epithelial cells (*n* = 4). (**B**) Concentration dependent increase of nuclear VDR by calcitriol over 24 h (*n* = 4). (**C**) The effect of combined EPs^®^ 7630 with calcitriol on the ratio of cytosolic versus nuclear VDR accumulation (*n* = 4) over 24 h. (**D**) The effect of MAPK inhibitors for JNK (SP600125), p38 (SB203580), and Erk1/2 (PD98059) on EPs^®^ 7630 and calcitriol induced VDR expression (*n* = 3). Bars represent mean ± SEM. Similar results were obtained in BEAS-2B cells.

EPs^®^ 7630 not only increased the overall expression of the VDR, but also induced its translocation into the nucleus (Figure 3A). However, this effect was much lower compared to calcitriol-induced nuclear accumulation of the VDR (Figure 3B). Both effects were concentration dependent. In untreated cells, the VDR was located mainly in the cytosol (Figure 3C). Calcitriol significantly increased the number of cells with nuclear VDR, and this was further increased when the cells were also treated with EPs^®^ 7630 (10 µg/mL) (Figure 3C). When cells were treated with the combination of EPs^®^ 7630 (10 µg/mL) and calcitriol, only Erk1/2 MAPK inhibition (PD98059) suppressed the accumulation of the VDR into the nucleus (Figure 3D).

### 2.3. Calcitriol Supports the Anti-Viral Effect of EPs^®^ 7630

**Figure 4:**
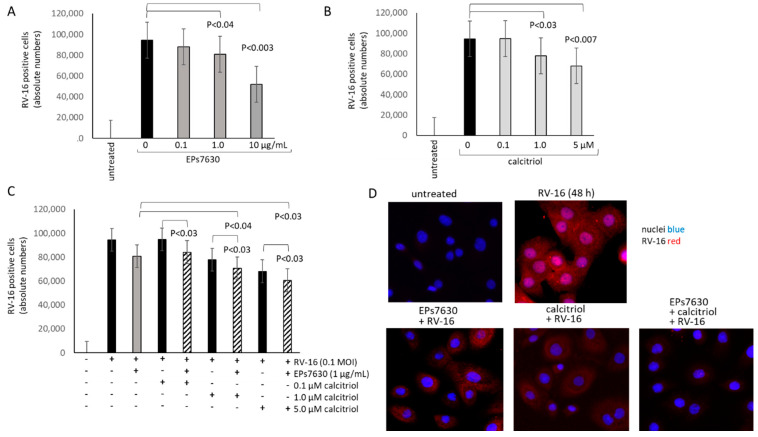
The effect of EPs^®^ 7630 and calcitriol on RV-16 infection in BEAS-2B cells. (**A**) Concentration dependent effect of EPs^®^ 7630 on RV-16 staining in BEAS-2B cells. (**B**) Concentration dependent effect of calcitriol on RV-16 staining in BEAS-2B cells. (**C**) The effect of combined EPs^®^ 7630 (fixed concentration) with calcitriol (increasing concentrations) on RV-16 staining on BEAS-2B cells. Bars represent mean ± SEM of triplicate experiments. (**D**) Representative immunofluorescence photographs of RV-16 positive primary epithelial cells in the presence and absence of EPs^®^ 7630 (1 µg/mL), or calcitriol (1 µM), or the combination of both (EPs^®^ 7630 1 µg/mL + calcitriol 1 µM).

In a previous study, EPs^®^ 7630 reduced the infection of epithelial cells by RV-16 [7]. This observation was confirmed by cell counting of the RV-positive stained BEAS-2B cells (Figure 4A). Calcitriol alone also reduced RV-infection of BEAS-2B cells in a concentration-dependent manner as shown in Figure 4B. When combined, the inhibitory effect of the two drugs on RV-16 infections was significantly improved, compared to calcitriol alone (Figure 4C). This effect was rather additive than synergistic. Representative immune-fluorescence photographs of the anti-viral effect of EPs^®^ 7630 in one primary epithelial cell line, with and without calcitriol are shown in Figure 4D.

### 2.4. EPs^®^ 7630 Increased Epithelial Cell Differentiation

**Figure 5:**
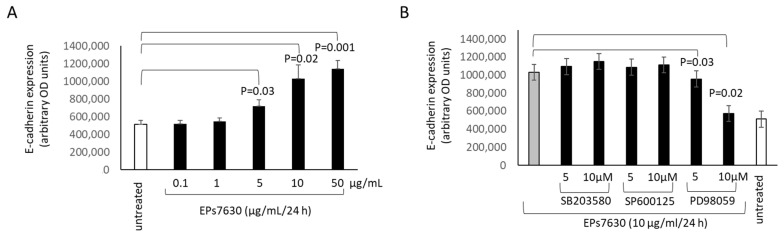
EPs^®^ 7630 increases E-cadherin expression through Erk1/2 MAPK in BEAS-2B cells. (**A**) Concentration effect of EPs^®^ 7630 on the expression of E-cadherin over 24 h in BEAS-2B cells. (**B**) The effect of inhibitors for JNK, p38, and Erk1/2 on EPs^®^ 7630 induced E-cadherin expression. Bars represent mean ± SEM of triplicate experiments.

E-cadherin is an indicator of epithelial cell maturation and is important for the cell-cell contact. Treating BEAS-2B cells with EPs^®^ 7630 over 24 h significantly increased the expression of E-cadherin in a concentration-dependent manner (Figure 5A). When inhibiting MAPKs, only PD98059, the inhibitor of Erk1/2, significantly reduced the expression of E-cadherin in BEAS-2B cells treated with EPs^®^ 7630 (Figure 5B). Similar results were obtained in one of the primary epithelial cell lines (data not shown).

## 3. Discussion

EPs^®^ 7630 enhanced the expression of the VDR in human bronchial epithelial cells through Erk1/2 MAPK signaling. Increased VDR expression improved its activation by calcitriol and might thereby enhance the anti-viral effect of EPs^®^ 7630.

The present study showed that EPs^®^ 7630 activates Erk1/2 MAPK signaling pathway, but inhibited p38 MAPK signaling, and had no effect on JNK. The data are in line with earlier studies in human monocytes [19]. Erk1/2 MAPK was the major regulating signaling protein for EPs^®^ 7630 induced expression of the VDR. This effect might occur through proanthocyanidins contained in EPs^®^ 7630 [22]. In human granulosa cells, proanthocyanidins increased the expression of Erk1/2 MAPK and of hormones at higher concentrations [23]. Thereby another proanthocyanidin, resveraterol, increased the expression of the VDR in other conditions [24]. However, other studies reported that proanthocyanidins and their metabolites inhibited Erk1/2, p38 and JNK MAPKs, and thereby reduced the reproduction of the hepatitis B virus [25]. These studies indicate that the effect of proanthocyanidins on different MAPKs might be concentration dependent, or varied with cell type or virus.

As displayed in Figure 1 and Table 1, EPs^®^ 7630 contains different forms of benzopyranones; little is known about the effect of this compound on the regulation of Erk1/2 signaling, VDR expression, and E-cadherin expression. Erk1/2 activation is essential for epithelial wound healing, proliferation and migration [26]. In line with our results, glycitein, a different benzopyranone, has been reported to upregulate the VDR in column cancer cells through Erk1/2 [27]. The role of Erk1/2 on the regulation of E-cadherin has mainly been studied in the context of epithelial-mesenchymal-transition. In this condition, Erk1/2 phosphorylation was inversely correlated with E-cadherin expression [28]. However, under different conditions such as hypoxia, epithelial cell differentiation involved the activation of Erk1/2, followed by E-cadherin expression [29]. Furthermore, E-cadherin has a negative feedback effect on Erk1/2 signaling in intestinal epithelial cells [30].

In addition, the expression of the VDR is linked to the expression of E-cadherin. In human bronchial epithelial cells (A549), vitamin D increased the expression of E-cadherin and thereby induced differentiation [31]. In gingival keratinocytes, vitamin D improved intercellular junctions by increasing E-cadherin expression [32]. The loss of VDR was associated with reduced expression of E-cadherin and epithelial cell differentiation in chronic kidney disease [33]. Thus, the literature indicates a mechanism linking VDR with E-cadherin and therefore epithelial cell differentiation with viral defense. One shortfall of this study is that Eps^®^ 7630 is a compound of various substances, therefore, we were not able to delineate the exact signaling mechanism.

The increased expression of the VDR by EPs^®^ 7630 might also account for the improved differentiation of epithelial cells by increasing their barrier function and host defense mechanisms. Low vitamin D levels were associated with increased epithelial de-differentiation and remodeling in COPD patients [34]. In gingival keratinocytes, the VDR action maintained the expression of E-cadherin and thereby reduced tissue remodeling [35]. In cancer cells, VDR activation prevented epithelial mesenchymal transition and thereby maintained the epithelial cell character [36]. However, the contribution of the VDR to epithelial cell differentiation might vary during the course of maturation [37].

In this study, EPs^®^7630 increased the expression of E-cadherin in non-differentiated bronchial epithelial cells by activating Erk1/2 MAPK. This observation is in line with the above-described EPs^®^ 7630 -induced activation of Erk1/2 MAPK in other cell types [19]. Future studies have to verify if EPs^®^ 7630 induced maturation of epithelial cells involves the VDR. In line with these results, E-cadherin expression was suppressed by RV-infection in a human bronchial epithelial cell line and resulted in EMT [38]. It was suggested that this mechanism might contribute to the thickness of the basal membrane in asthma. Furthermore, RV infection delayed the repair of TGF-β-induced epithelium injury, which correlated with reduced epithelial cell differentiation, and low E-cadherin expression [39].

In conclusion, an increase of E-cadherin expression indicates that EPs^®^ 7630 might reduce the thickening of the airway wall in airway infection or inflammation. The upregulation of the VDR and the improved response to vitamin D in EPs^®^ 7630 treated epithelial cells supports the anti-viral effect of the compound. However, this effect has to be confirmed by clinical studies and in animal models.

## 4. Materials and Methods

### 4.1. Primary Bronchial Epithelial Cells and BEAS-2B Cell

Primary bronchial epithelial were isolated and characterized as described earlier [32]. Endo-bronchial tissue biopsies were obtained from six patients by endo-bronchoscopy for diagnostic reasons by the lung clinic (University Hospital Basel, Switzerland). All patients provided written informed consent for the use of one additional anonymous biopsy for scientific investigations. The study was approved by the local Institutional Ethical Committee (EKBB 05/06).

**Figure 6:**
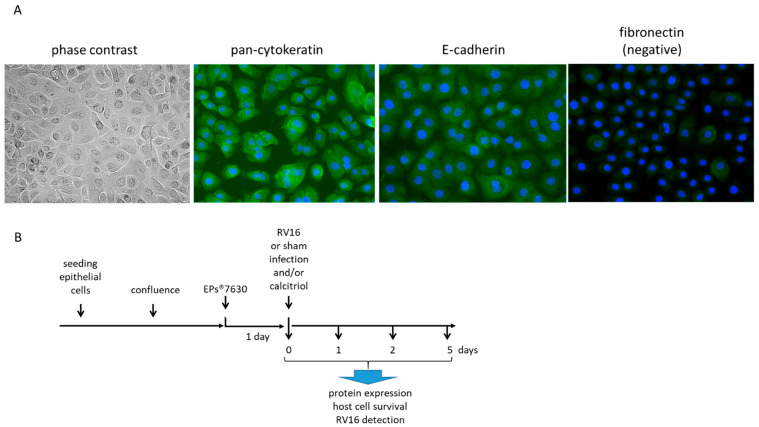
Cell characterization and treatment. (**A**) Cell type characterization by phase contrast microscopy and positive immunofluorescence pan-cytokeratin, E-cadherin, and fibronectin (negative control). (**B**) Treatment scheme of cells. Confluent epithelial cells were pre-treated with either EPs^®^ 7630 for 24 h before calcitriol was added for another 24 h and prior to infection with 1 MOI RV-16. RNA and protein were isolated over 4 days. Non-infected cells were used to calculate changes of protein expression at the corresponding time points.

Epithelial cells were isolated by cell-type selective medium CnT-PR-A (CellnTec, Bern, Switzerland). BEAS-2B cells were grown in the same cell culture medium. The cell phenotype was monitored by phase contrast microscopy and by staining for: positive E-cadherin and cytokeratin-13, as well as for negative fibronectin staining (Figure 6A).

### 4.2. Drugs

EPs^®^ 7630, a herbal drug preparation from the roots of *P. sidoides* (1:8–10), extraction solvent: ethanol 11% (*w/w*). The concentration of EPs^®^ 7630 is 60 mg/day, which is equivalent to 1 μg/kg for an average person, or to 1 μg/mL. The concentrations used for this study ranged between 0.1–100 μg/mL, with a single application at day 0. EPs^®^ 7630 was supplied as dry powder by Schwabe Pharma AG (Karlsruhe, Germany) and was dissolved in cell culture medium to the desired end-concentration.

Vitamin D, calcitriol (Sigma-Aldrich Merck, Buchs, Switzerland), was dissolved in ethanol (100 μg/mL) before being added to the cell cultures at final concentrations ranging from concentrations of 0.1–10 μM, together with EPs^®^ 7630.

### 4.3. High Pressure Liquid Chromatography

EPs^®^ 7630 high pressure liquid chromatography-UV high resolution mass spectrometry (HRMS): The HPLC-UV-HRMS chromatograms were recorded on an Thermo^®^ Vanquish UHPLC coupled to a DAD and Thermo Orbitrap^®^ Fusion mass detector using a Waters^®^ Atlantis T3 (3 µM, 2 mm × 150 mm) column without pre-column. Eluent A consisted of 2.5% (*v/v*) acetonitrile and 0.5% (*v/v*) formic acid in water. Eluent B consisted of 5% (*v*/*v*) water and 0.5% (*v/v*) formic acid in acetonitrile.

At a flow rate of 0.2 mL/min, the gradient was as follows: from 0.0–10.0 min linear for 0% to 5% eluent B, from 10.0–65.0 min linear for 5% to 50% eluent B, from 65.0 to 66.0 min linear for 50% to 100% eluent B, from 66.0 to 71.0 min isocratic 100% eluent B column wash, from 71.0–72.0 min linear from 100% to 0% eluent B followed by 8 min equilibration period with 0% eluent B, resulting in a total run time of 80.00 min. UV detection wavelength of 280 nm and a column temperature of 40 °C were applied. The injection volume was 4 µL of a 5 mg/mL *P. sidoides* extract EPs^®^ 7630 dissolved in eluent A. HRMS based peak assignment was performed using ACDLabs Spectrus Processor Software v2017.2.1.

### 4.4. Cell Treatment

Confluent epithelial cells were stimulated with EPs^®^ 7630 (0.1–10 µg/mL), or calcitriol, or the combination of both, for up to 48 h before being infected with 1 MOI of RV-16 as depicted in Figure 6B.

For EPs^®^ 7630-induced VDR expression, cells were pre-treated for 24 or 48 h with EPs^®^ 7630 (10 µg/mL). To determine the effect of EPs^®^ 7630 and calcitriol on VDR translocation pre-treated cells were exposed to increasing concentration of vitamin D (calcitriol 0.1–10 μM in DMSO). The expression of the VDR was determined by Western-blotting in total protein extracts. In some experiments, the EPs^®^ 7630 pretreated cells were infected with 1 MOI RV-16 for up to 48 h. Total proteins were collected over 4 days and analyzed as described below for protein expression, or cells grown on cover slips were fixed with 2% formaldehyde for 2 × 5 min. followed by staining and fluorescence microscopy for RV16 protein expression (Figure 6B).

### 4.5. Rhinovirus Infection and Detection

The RV-16 strain was purchased from ATCC (RV-16, VR283, American Type Culture Collection, Manassas, VA, USA). Cells were seeded in 8-well chamber slides (Thermo Fisher Scientific, Basel, Switzerland) and at 80–90% confluence they were infected with RV16 (1× multiplicity of infection: MOI) by 5 min centrifugation (200× *g*). Cells were continued under standard cell culture conditions for up to 4 days. RV16 infection was monitored by immunofluorescence for anti-RV16 antibody (cat# 18758, QED-Bioscience Inc., San Diego, CA, USA). Cells were fixed by formalin (4% in PBS, 2 × 5 min.), washed twice (PBS), and permeabilized (5 min., 0.01% TWEEN-100 in PBS). Unspecific antibody binding was blocked (30 min., 2% bovine serum albumin in PBS) and afterwards incubated with the anti-RV16 antibody (1:100 dilution, overnight, (4 °C). Cells were washed 3× (PBS), followed by incubation with anti-mouse FITC labelled antibody (Abcam, Switzerland, 1 h, room temperature). Nuclei were stained by DAPI for cell counting (Thermo Fisher Scientific). The number of RV16 positive cells was determined after 3 washes (PBS) by immunofluorescence microscopy (EVOS FLoid cell imaging station, Thermo Fisher Scientific). All experiments were performed in a Bio-Safety-Level-II laboratory.

### 4.6. Cytosolic—Nuclear Protein Translocation

Confluent epithelial cells were treated with EPs^®^ 7630 (10 µg/mL) for 48 h before being stimulated with increasing concentrations of vitamin D (0.1–10 mM) over various time periods (0, 3, 6, and 24 h). The cell compartmental distribution of the VDR was determined by immunofluorescence staining using EVOS cell imaging system (Thermo Fisher Scientific).

### 4.7. Western-Blotting

Cells were lysed in RIPA buffer, or as cytosolic and nuclear proteins. The protein content was quantified by BCA (Thermo Fisher Scientific). Denatured proteins (10 µg) were size-fractionated by electrophoresis (8–16% SDS–PAGE, Thermo Fisher Scientific), and transferred onto PVDF membranes. Unspecific binding of antibodies was blocked by 30 min incubation of the membranes with 2% bovine serum albumin in phosphate buffer saline (PBS) containing 0.05% TWEEN-20. Proteins were detected by incubating the membranes with one of the primary antibodies to either the VDR, Erk1/2 MAPK, p38 (α, β, γ, δ), JNK, E-cadherin, or GAPDH (all: Abcam Plc, Cambridge, UK) for overnight at 4oC. Following three washes with blocking buffer, the membranes were incubated with secondary species-specific HRP conjugated antibodies (Abcam). Protein bands were visualized by chemiluminescence, applying SuperSignal West Dura substrate (Thermo Fisher Scientific) and documented by c300 (Azure Biosystems, Dublin, CA, USA).

### 4.8. Immunofluorescence

Epithelial cells were seeded on 8-well PCA-slides (cat 94.6140.802, Sarstedt, Sevelen, Switzerland) and allowed to adhere overnight. Cells were then treated with medium alone, or by increasing concentrations of EPs^®^ 7630 (0.01–10 µg/mL) over various durations up to five days. Cells were fixed in 4% paraformaldehyde (in PBS, 2 ×5 min), and immuno-fluorescence staining was performed as described earlier [7]. Nuclei were stained by DAPI.

### 4.9. Statistics

The Null-hypothesis was: no modification of protein expression, activation or location by any treatment compared to untreated cells. The Null-hypothesis was tested by ANOVA, Student’s t-test and subsequent Mann-Whitney U-test as appropriate; *p*-value < 0.05 was accepted as significant.

## 5. Conclusions

This study indicates that the protective effect of EPs^®^ 7630 against rhinovirus infection might involve the upregulation of the VDR and the improved epithelial cell differentiation.

## Figures and Tables

**Table 1:** Benzopyranones of EPs^®^ 7630 as analyzed by HPLC-UV-HRMS (see Figure 1).

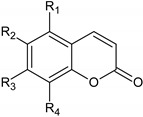					
Assigned Peak in Figure 1	Name	R_1_	R_2_	R_3_	R_4_
d	6,8-bissulfooxy-7-hydroxy-2*H*-1-benzopyran-2-one	H	SO_4_^−^	OH	SO_4_^−^
e	6,7-dihydroxy-8sulfooxy-2*H*-1-benzopyran-2-one	H	OH	OH	SO_4_^−^
f	7,8-dihydroxy-6-sulfooxy-2*H*-1-benzopyran-2-one	H	SO_4_^−^	OH	OH
g	8-hydroxy-7-methoxy-6-(sulfooxy)-2*H*-1-benzopyran-2-one	H	SO_4_^−^	OCH_3_	OH
h	6-methoxy-7-sulfooxy-2*H*-1-benzopyran-2-one	H	OCH_3_	SO_4_^−^	H
k	5,6-dimethoxy-7,8-dihydroxy-2*H*-1-benzopyran-2-one	OCH_3_	OCH_3_	OH	OH
m	7-hydroxy-5,6-dimethoxy-8-sulfooxy-2*H*-1-benzopyran-2-one	OCH_3_	OCH_3_	OH	SO_4_^−^
n	5,6-dimethoxy-7-sulfooxy-2*H*-1-benzopyran-2-one (Umckalin-7-sulphate)	OCH_3_	OCH_3_	SO_4_^−^	H
o	7-hydroxy-5,6-dimethoxy-2*H*-1-benzopyran-2-one (Umckalin)	OCH_3_	OCH_3_	OH	H

## Data Availability

The data sets used and/or analyzed during the current study are available from the corresponding author on reasonable request.
